# Investigation of directionally solidified InGaSb ternary alloys from Ga and Sb faces of GaSb(111) under prolonged microgravity at the International Space Station

**DOI:** 10.1038/npjmgrav.2016.26

**Published:** 2016-07-21

**Authors:** Velu Nirmal Kumar, Mukannan Arivanandhan, Govindasamy Rajesh, Tadanobu Koyama, Yoshimi Momose, Kaoruho Sakata, Tetsuo Ozawa, Yasunori Okano, Yuko Inatomi, Yasuhiro Hayakawa

**Affiliations:** 1 Research Institute of Electronics, Shizuoka University, Hamamatsu, Japan; 2 Graduate School of Science and Technology, Shizuoka University, Hamamatsu, Japan; 3 Centre for Nanoscience and Technology, Anna University, Chennai, India; 4 Department of Chemical System Engineering, University of Tokyo, Chiba, Japan; 5 Department of Electrical Engineering, Shizuoka Institute of Science and Technology, Shizuoka, Japan; 6 Graduate School of Engineering Science, Osaka University, Osaka, Japan; 7 Institute of Space and Astronautical Science, Japan Aerospace Exploration Agency, Kanagawa, Japan; 8 School of Physical Sciences, SOKENDAI (The Graduate University for Advanced Studies), Kanagawa, Japan

## Abstract

InGaSb ternary alloys were grown from GaSb (111)A and B faces (Ga and Sb faces) under microgravity conditions on board the International Space Station by a vertical gradient freezing method. The dissolution process of the Ga and Sb faces of GaSb and orientation-dependent growth properties of InGaSb were analysed. The dissolution of GaSb(111)B was greater than that of (111)A, which was found from the remaining undissolved seed and feed crystals. The higher dissolution of the Sb face was explained based on the number of atoms at that face, and its bonding with the next atomic layer. The growth interface shape was almost flat in both cases. The indium composition in both InGaSb samples was uniform in the radial direction and it gradually decreased along the growth direction because of segregation. The growth rate of InGaSb from GaSb (111)B was found to be higher than that of GaSb (111)A because of the higher dissolution of GaSb (111)B.

## Introduction

High-quality crystals with tuneable properties are desirable as substrate materials for various electronic and optoelectronic device applications.^1,2^ InGaSb ternary alloys have tuneable physical properties between their binary counterparts InSb and GaSb in the infrared region; hence, they are suitable for making thermophotovoltaic (TPV) devices and infrared detectors.^3–6^ Although InGaSb has been identified as a potential material for making infrared devices and in TPV applications, growth of high-quality InGaSb crystals, over a wide range of indium composition, is a difficult task.^7,8^ A large gap between the liquidus and solidus lines of the InSb–GaSb binary phase diagram results in segregation of the compound.^9,10^ Moreover, constitutional super cooling occurs during the solidification process when the gradient of the composition exceeds the critical value at the solid–liquid interface, which means that graded composition and interface breakdown can easily occur in the grown crystal.^11^ Hence, it is crucial to understand the growth properties to overcome these difficulties that arise in the growth of ternary alloys. In addition to these factors, the convection process in a melt driven by gravity would affect the heat and mass transport processes, changing the kinetics of growth at various stages.^12^

Growth of high-quality ternary alloys for device applications requires pre-knowledge of growth parameters, such as dissolution, mass and heat transport, and the kinetics involved in growth. Hence, to understand heat and mass transport phenomena, a gravity-free environment for long durations in space is required.^13,14^ With this aim, numerous microgravity experiments have been performed in space flights, recoverable satellites, and space shuttles to understand the effect of gravity on the growth process. Some of these experimental results were unexpected because of the limitations of the duration of the microgravity environment, which was not sufficient to carry out the growth of large bulk crystals.^15^ Our group has also carried out microgravity experiments in a drop tower, space shuttle, and Chinese recoverable satellite in which the formation of a projection during solidification, needle crystal formation, growth morphology, composition distribution, and melt mixing were explained.^16–18^ We analysed the effect of substrate orientation on the morphological change of the solid–liquid interface, and the orientation dependence of the step kinetic coefficient at the GaP/GaP interface was reported earlier.^19^ A numerical model was developed to evaluate the effect of crystal orientation on the growth rate.^20^

The International Space Station (ISS) provides prolonged microgravity conditions for materials production, space medicine, plant biology, biotechnology, and communications, which have been explored since 2000. Significant results in the fields of cell biology, plant architecture, human physiology, and materials science have been reported utilizing the space platform “ISS”.^21–24^ To utilize the facilities provided by ISS, in collaboration with Japan aerospace exploration agency, we were making a sequence of experiments to understand the orientation-dependent growth properties of InGaSb ternary alloy along different primary planes of GaSb (111) [A and B], (110) and (100), under microgravity at ISS as well as on Earth. The crystal growth experiments under microgravity were carried out in the Japanese space experimental module “KIBO”, a kind of space lab, installed in the ISS.

The sequence of experiments (Alloy semiconductor project) involved four experiments on board ISS and four experiments on Earth, totally eight similar experiments under microgravity and terrestrial conditions. We were carefully analyzing and reporting the experimental results one-by-one, as this was a complete significant experiment to understand the orientation-dependent dissolution and growth process of a ternary alloy, probably a first of this kind that could expect to reveal important results in the field of “microgravity materials science”, in particular “crystal growth” experiments. The eight experiments of this alloy semiconductor project were done at various time periods between 2010 and 2015. We were carrying out the first phase analysis of the samples in a step-by-step manner that was started from 2013. The first experimental results of this project is a comparative study between the properties of InGaSb crystals grown from GaSb(111)A under microgravity and terrestrial condition in which the phenomena of higher growth rate and lower dislocation density in microgravity under suppressed convective and dominant diffusive transport were explained.^25^

In the present work, we explain the orientation-dependent growth properties of InGaSb ternary alloys grown along the GaSb (111)A and (111)B faces (i.e., Ga and Sb faces) by a vertical gradient freezing (VGF) method under prolonged microgravity condition at ISS.

## Results

**Figure 1:**
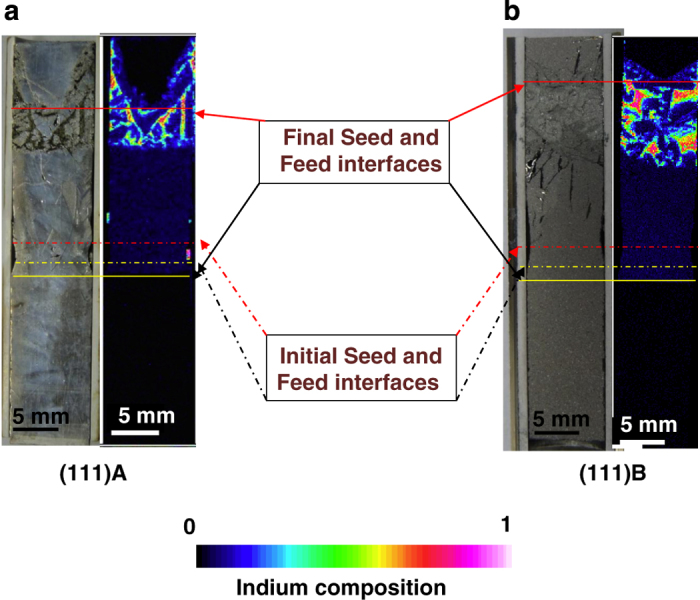
Cross section and indium composition mapping of InGaSb crystals grown along GaSb (**a**) (111)A and (**b**) (111)B faces. The red and yellow lines show the seed and feed interfaces at initial (dotted line) and final (continued line) stages of growth.

The seed interface temperatures of (111)A and (111)B experiments, at the earlier stage of growth, were found to be 687.3 and 688.2 °C, respectively, and the temperature gradients were calculated to be 0.81 and 0.90 °C/mm, respectively, from the recorded temperature profile. The (111)B experiment had 11.1 % higher temperature gradient than (111)A. The InGaSb samples were cut along the (110) plane, and their electron probe micro analysis (EPMA) mapping for indium distribution with their initial and final seed and feed interfaces are shown in Figure 1a,b. (Hereafter, InGaSb grown along the (111)A and (111)B faces are denoted (111)A and (111)B samples, respectively). The dissolution lengths of the seed and feed crystals of both (111)A and (111)B samples were calculated from the remaining undissolved crystals. A total of 2.3 and 2.5 mm of seed crystals, and 14.4 and 19.9 mm of feed crystals, were dissolved in (111)A and (111)B samples, respectively. The initial growth (seed) interface for both (111)A and (111)B samples was almost flat, whereas the feed crystals remained in a “V” shape. Hence, the dissolution length of the feed crystal was considered in the middle position rather than the periphery, where more GaSb was dissolved. The remaining indium-rich solution was solidified during the cooling process near the feed crystal in both samples.

**Figure 2:**
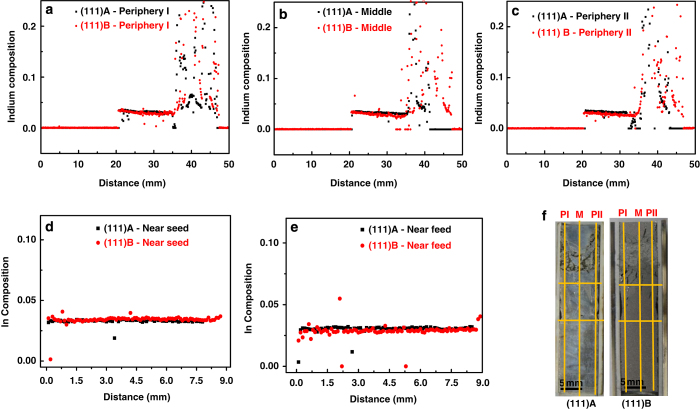
Indium composition measured by EPMA along the vertical directions (**a**) periphery—I, (**b**) middle, and (**c**) periphery—II; and radial directions near the (**d**) seed interface and (**e**) feed interface; and (**f**) measured positions of InGaSb crystal surfaces.

The vertical distribution of indium composition was measured at three positions (Periphery—I, Middle and Periphery—II) along the growth direction, and the radial distribution was measured at two positions, near the seed and in the middle position. Figure 2 shows the indium composition along the vertical (Figure 2a,c) and radial (Figure 2d,e) directions for both (111)A and (111)B samples, along with the measured position of the crystals (Figure 2f). The initial indium composition for both the samples was ~0.034 and gradually decreased to 0.030 in the (111)A sample, whereas it ended up at 0.026 in the (111)B sample. The radial distribution of indium was uniform in both samples.

**Figure 3:**
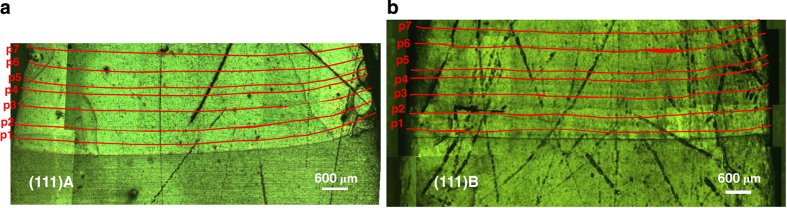
Initial seed interface shape and striations of (**a**) (111)A and (**b**) (111)B samples.

**Figure 4:**
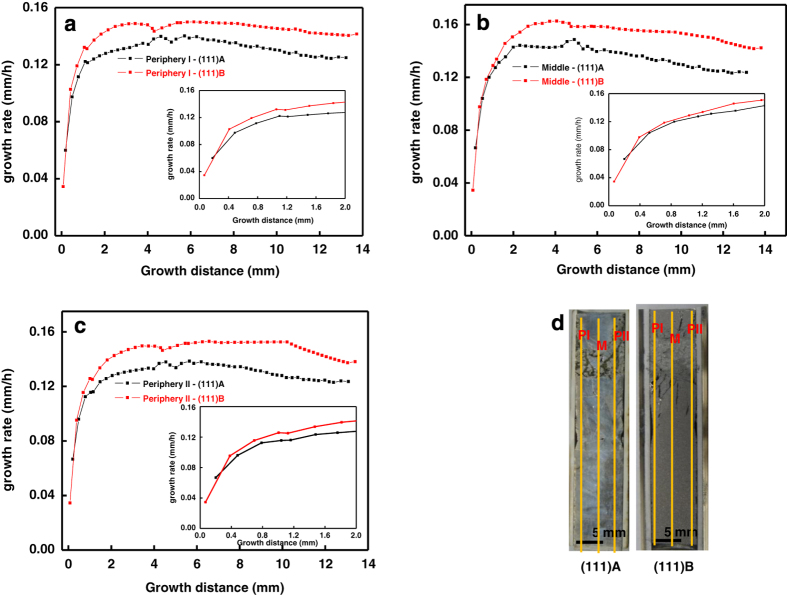
Growth rates of InGaSb along the (**a**) periphery—I, (**b**) middle, and (**c**) periphery—II; and (**d**) crystal surface showing the measured positions.

The etched surfaces of the (111)A and (111)B samples near the growth interface are shown in Figure 3 in which the striations are marked with red lines for clear visibility. The initial growth interface shape was almost flat in both samples, and the striations had a flat interface. The growth rates of the (111)A and (111)B samples were calculated along three vertical positions (Periphery—I, Middle and Periphery—II) by measuring the distances between the induced growth striations. Figure 4 shows the growth rate profiles along three positions: periphery—I (Figure 4a), middle (Figure 4b), and periphery—II (Figure 4c) of (111)A and (111)B samples along with the calculated positions (Figure 4d) of the crystals. The insets of the figures show the initial stage of growth at the corresponding positions. The growth rates at the saturated end (later stage of growth) of (111)A and (111)B samples were 0.13 and 0.15 mm/h, respectively.

## Discussion

**Figure 5:**
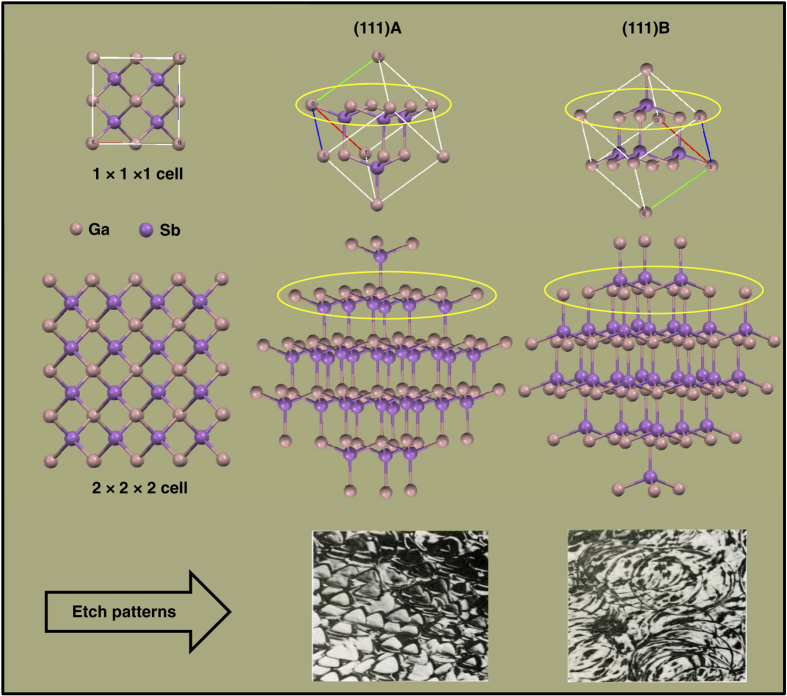
Atomic arrangement and etch patterns of GaSb (111)A and (111)B faces.

From the dissolution lengths of the seed and feed crystals, the dissolution of (111)B was found to be greater than that of (111)A because of the different arrangement of atomic layers. The higher dissolution of the Sb face can be explained based on the atomic arrangement and their binding with the next layer of that plane. The atomic arrangement of GaSb 1×1×1 cell and 2×2×2 cells along the (111)A and (111)B faces are shown in Figure 5. The (111)A face has the arrangement Ga–Sb–Ga–Sb, and the (111)B face has Sb–Ga–Sb–Ga repeated atomic layers. From the figure, it is clear that in a GaSb 1×1×1 cell, the (111)A face has six Ga atoms, three at corner and three at face-centered positions. The corner Ga atoms were bonded with the next Sb layer with a single bond, whereas the face-centered Ga atoms had two bonds with the Sb atoms. In total, six Ga atoms were bonded with the next layer, which has three Sb atoms, by nine bonds. For the (111)B face, a single Sb atom was bonded with the next layer, which has six Ga atoms in which three face-centered Ga atoms bond with that Sb atom. Considering GaSb 2×2×2 cells, ten Ga atoms were bonded with the next atomic layer, which has six Sb atoms, in the (111)A face, whereas in the (111)B face, three Sb atoms were bonded with seven of ten Ga atoms in the next atomic layer.

The number of atoms in each atomic layer and their bonds with the next layer were calculated for 1×1×1 and 2×2×2 cells. The calculated number of atoms and bonds in GaSb (111)A and (111)B faces are given in Table 1. The total number of Ga and Sb atoms in the (111)A and (111)B faces were the same for 1×1×1 and 2×2×2 cells, whereas the binding between the atoms shows a significant difference. For the 1×1×1 cell, the (111)A face had 16 bonds, whereas the (111)B face had 15 bonds. In the case of 2×2×2 cells, the (111)A and (111)B faces had a total number of 124 and 122 bonds, respectively. This shows that the 1×1×1 cell had one excess bond and the 2×2×2 cells had two excess bonds in their (111)A face. As the number of unit cells increased, the excess bonds in the (111)A face increased. When we consider a bulk material of *n*×*n*×*n* cells, it would have *n* number of excess bonds in (111)A compared with the (111)B face. Because there are more bonds, more activation energy is required to break the bonds in the (111)A face compared with the (111)B face; hence, the dissolution of (111)B was observed to be greater. The obtained result was consistent with our previous microgravity experiment.^17^

The dissolution of GaSb seed crystals stopped when supersaturation was attained at the interface and the crystal started to grow. The indium composition along the vertical direction (Figure 2a,c) measured by EPMA shows that it gradually decreased along the growth direction. During the growth process, the growth interface moved towards the high-temperature feed region because the temperature gradient was maintained in the furnace. Hence, the indium composition along the growth region decreased according to the phase diagram. Because convection was suppressed under microgravity, the growth process was diffusion-controlled and the composition measured along the radial direction shows a uniform distribution of indium in the grown crystals. The random distribution of indium in the later stages of growth occurred because of the solidification of residual melt when cooling was applied to the system.

The growth rates (Figure 4) at various positions along the growth direction indicate that the (111)B face had a higher growth rate than the (111)A face. Even though the growth rate was observed to be higher for the (111)B sample, the difference between the growth rates was minimum at the initial stage, which would be within the error limit. Hence, at the initial stage, we might not conclude that the growth rate for (111)B was high. However, a higher growth rate was clearly observed in the later stages of the experiment. Moreover, the differences between the growth rates increased from the initial to the final stage, which can be clearly seen in Figure 4. It was found that the growth rate of the (111)B face was 15.4 % higher than that of (111)A.

Considering that the growth under microgravity by VGF is a diffusion-controlled steady state process and assuming the solute was saturated in the solution, the relationship between the growth rate and solute concentration is given by,^26^

(1)V=−D(Cl0−Cs0)(∂CL∂Z)Z=0=−D(Cl0−Cs0)(∂CL∂T)(∂T∂Z)Z=0,

where *D*=inter diffusion coefficient between solute and solvent (diffusion coefficient of GaSb under microgravity); $\frac{\partial C_{L}}{\partial Z}$= composition gradient in solution along the distance* z*; $\frac{\partial C_{L}}{\partial T}$=reciprocal of the slope of the liquidus line in the InSb–GaSb binary phase diagram; $\frac{\partial T}{\partial Z}$=temperature gradient in the solution (i.e., temperature gradient applied to the furnace); and *C*_*l*0_, *C*_*s*0_=GaSb concentration in solution and that in the crystal at the growth interface, respectively.

In equation (1), the terms D, *C*_*l*0_, and *C*_*s*0_ are constants. The growth rate was observed to be higher for InGaSb grown from GaSb (111)B than would be extrapolated from the results for (111)A (Figure 4) based on a direct proportionality to concentration gradient, $\frac{\partial C_{L}}{\partial Z}$ in melt. That means the higher dissolution of GaSb should also be evaluated as a cause of the higher growth rate of InGaSb. In the present experiment, the temperature gradient $\left(\frac{\partial T}{\partial Z}\right)$ was attempted to be fixed as a constant, but in the actual case, the (111)B experiment had a temperature gradient 0.09 °C/mm higher than that of (111)A. It becomes necessary to find out which factor, either the concentration of melt or the 0.09 °C/mm higher temperature gradient caused the higher growth rate of (111)B.

Hence, it was necessary to analyse the effect of the temperature gradient on the growth rates of (111)A and (111)B samples. To accomplish this, the concentration gradient term $\frac{\partial C_{L}}{\partial T}$ was assumed to be constant for both (111)A and (111)B experiments. Then, equation (1) can be rewritten as,

(2)V=A(∂T∂Z)Z=0,

where *A*=$-\frac{D}{\left(C_{l0}-C_{s0}\right)}\left(\frac{\partial C_{L}}{\partial T}\right)$ is a constant.

On the basis of the equation (2), the growth rate was directly proportional to the temperature gradient.

Let *V*_1_ and *V*_2_ be the growth rates and ∂*T*_1_/∂Z and ∂*T*_2_/∂Z be the temperature gradients of the (111)A and (111)B experiments, respectively. Then, the growth rates of the (111)A and (111)B experiments are,

(3)V1=A(∂T1∂Z),

(4)V2=A(∂T2∂Z),

(5)V1V2=∂T1/∂Z∂T2/∂Z.

The above equation shows that the ratio between growth rates should be equal to the ratio between the temperature gradients of the (111)A and (111)B experiments. The experimental results showed that (111)B had a 15.4 % higher growth rate and 11.1 % higher temperature gradient than (111)A. The ratio of growth rate was higher than that of the temperature gradient. Hence, it is clear that the higher growth rate of the (111)B experiment was not only influenced by the temperature gradient $\left(\frac{\partial T}{\partial Z}\right)$, but also by another factor $\left(\frac{\partial C_{L}}{\partial T}\right)$. The factor $\frac{\partial C_{L}}{\partial T}$ depends on the dissolution of the GaSb feed. The dissolution of the GaSb feed was found to be higher in (111)B than (111)A (Figure 1). Hence, the greater amount of solute in the solution resulted in the higher growth rate of (111)B compared with (111)A because of the higher concentration gradient $\left(\frac{\partial C_{L}}{\partial T}\right)$.

InGaSb crystals were grown from the Ga and Sb faces of GaSb (111) under prolonged microgravity at the ISS for 230 h by a VGF method, which was the first experiment of its kind, to study the orientation-dependent dissolution and growth properties of a ternary alloy under microgravity. The temperature profiles used for both experiments were similar, and the temperature gradient varied slightly; i.e., it was 0.09 °C/mm higher in the (111)B experiment than in the (111)A experiment. The experimental results indicate that the GaSb seed and feed crystals dissolved more along (111)B than (111)A because of the difference in the atomic arrangement of Ga and Sb atoms and their binding with the next atomic layer in their respective planes. The indium composition along the growth direction gradually decreased from the low-temperature seed interface to the high-temperature feed interface according to the InSb–GaSb phase diagram. The indium composition across growth was homogeneous, showing the uniform distribution of the temperature profile along the radial direction. The distance between growth striations was measured to calculate the growth rate and it was found that the difference between the growth rates at the initial stage was very small when compared with the later stages of growth. In a diffusion-controlled growth process under microgravity, the dissolution of GaSb (111)B was higher than that of (111)A and the growth rate of InGaSb ternary alloy from GaSb (111)B was greater than that of GaSb (111)A.

## Materials and methods

**Figure 6:**
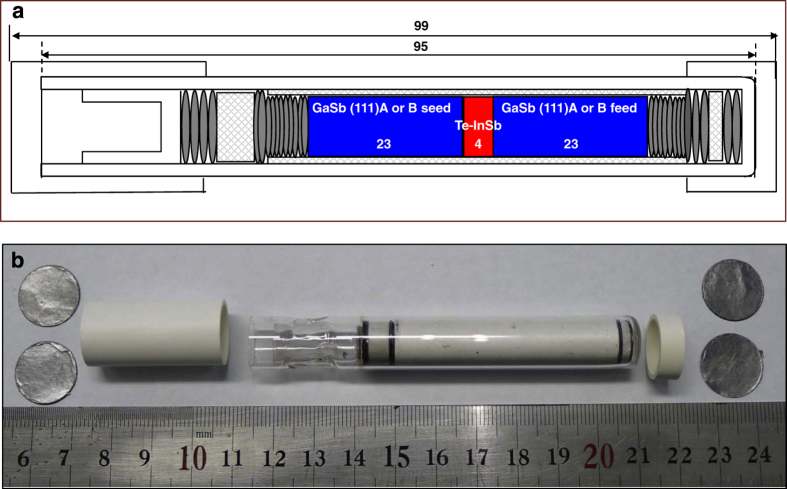
(**a**) Schematic of ampoule design and (**b**) prepared ampoule with sandwich structure of crystals GaSb(111)A or B seed/Te-doped InSb/GaSb (111)A or B feed.

InGaSb ternary alloys were grown along the (111)A and (111)B faces of GaSb by the VGF method using sandwich-structured ampoules of GaSb(111)A/InSb/GaSb(111)A and GaSb(111)B/InSb/GaSb(111)B. The ampoule was specifically designed to protect the crystals before and after the experiment to withstand the vibration associated with the space environment. The vibration can originate from the rocket launch and return of samples to Earth. The schematic design for the preparation of the ampoule is shown in Figure 6a. A GaSb single crystal along the (111) plane was grown by the Czochrolski method and was cut and polished using SiC and alumina abrasives of different particle sizes to reduce the diameter, then shaped in a lathe machine to obtain cylindrical chunks with dimensions of 23×9 mm. The Ga and Sb faces of the (111) plane were identified from their etch patterns, which show triangular and circular etch pits, respectively. The GaSb unit cell, the atomic arrangement of Ga and Sb faces, and the etch patterns of the (111)A and (111)B planes are shown in Figure 5. The cylindrical GaSb single crystals grown along the (111) plane were packed with InSb poly crystals (dimensions: 4×9 mm) in a boron nitride (BN) tube along with carbon sheets and BN disks. The carbon sheets were added to adjust the volume change during solid–liquid and liquid–solid phase transitions; they also act like a spring to withstand the vibration before and after the experiment while sending to the ISS and returning to Earth. Before ampoule preparation, a wettability test of GaSb, InSb, and In*_x_*Ga_1−*x*_Sb materials was performed with the ampoule packing materials, BN, carbon sheet, quartz, and C-103 alloy (cartridge material) at higher temperatures to check the compatibility of these materials during the growth process.^27^ The crystals were packed according to the ampoule design, under nitrogen flowing conditions, to maintain an inert atmosphere inside the ampoule, which was evacuated up to 10^−4^ Pa using a turbo molecular pump. The evacuation process was continued for 24 h, and the ampoule was sealed off at this state. Two ampoules, with GaSb(111)A/InSb/GaSb(111)A, and GaSb(111)B/InSb/GaSb(111)B structures, were made for the experiment, and an image of a prepared ampoule is shown in Figure 6b.

The prepared ampoules were loaded into the furnace cartridges, which were sent to the ISS earlier, in 2011. The InGaSb growth experiment along the GaSb (111)A and (111)B faces was carried out with a duration of 230 h, which was sufficient to grow a bulk crystal in a steady state condition under prolonged microgravity at the ISS. This is the first experiment of this kind, carried out under prolonged microgravity, to study the orientation-dependent growth of ternary alloy semiconductors.

The growth experiments were performed in a gradient heating furnace by the VGF method. The technique we used for directional solidification of InGaSb was explained in our previous article.^25^ For both (111)A and (111)B experiments, similar heating profiles and heat pulses were applied during the growth process. The heat pulses were applied to induce striations in the grown crystals, which would give solid–liquid interface shapes and growth rates at various time periods. The temperature inside the cartridge was measured and recorded by five equidistant thermocouples positioned at 21-mm intervals, covering the whole ampoule height, which was adequate to monitor and record the temperature at various positions of the ampoule during the growth experiment. For the growth experiment, the temperature of the furnace was increased up to 700 °C at a heating rate of 0.2 °C/min and it was held for ~100 h at this temperature. Heat pulses were applied during this stage. After applying heat pulses, the furnace was cooled down slowly at a rate of 0.5 °C/min to 400 °C, and then a 1 °C/min cooling rate was applied until room temperature was reached.

After the growth experiments, the cartridges were returned to Earth by a Russian rocket. The ampoule was removed from the cartridge by cutting it using a pressurized water jet. The grown crystals were cut into two halves along the (110) plane (i.e., along the growth direction) using a diamond saw. For the analyses, one half of the cut crystal was polished using SiC and alumina abrasives of different particle sizes to obtain a mirror-finish surface. The polished crystals were etched in a 1:1:1 ratio of HF:HNO_3_:CH_3_COOH etchant to remove impurities from the surface, and the indium composition was analysed by EPMA. The growth striations were observed by etching in a 1:3:1 ratio of HF:KMnO_4_:CH_3_COOH etchant for 30 min. The striation images were captured using an optical interference microscope; the images were used to calculate the distance between striations and growth rates.

## Figures and Tables

**Table 1:** Number of atoms and bonds in the (111)A and (111)B faces of GaSb 1×1×1 cell and 2×2×2 cells

*Atomic layer*	*(111)A*			*(111)B*		
	*No. of atoms*		*No. of bonds with next atomic layer*	*No. of atoms*		*No. of bonds with next atomic layer*
	*Ga*	*Sb*		*Sb*	*Ga*	
*GaSb*—*1×1×1 cell*						
1st	6		9	1		3
2nd		3	3		6	3
3rd	6		3	3		9
4th		1	1		6	0
Total	12	4	16	4	12	15
						
*GaSb*—*2×2×2 cells*						
1st	10		18	3		9
2nd		6	6		10	10
3rd	18		36	10		30
4th		12	12		18	12
5th	18		30	12		36
6th		10	10		18	6
7th	10		9	6		18
8th		3	3		10	1
Total	56	31	124	31	56	122
